# Supplementary material for: Nanostructured Scaffold, Combined with Human Dental Pulp Stem Cell Secretome, Induces Vascularization in Medicinal Leech Model
Source: Micromachines (Basel). 2025 Oct 10;16(10):1150. doi: 10.3390/mi16101150 (PMC12565914; doi:10.3390/mi16101150)
Supplement: Supplementary file 1 [file micromachines-16-01150-s001.zip › micromachines-3883225-supplementary.pdf]

Supplementary Materials

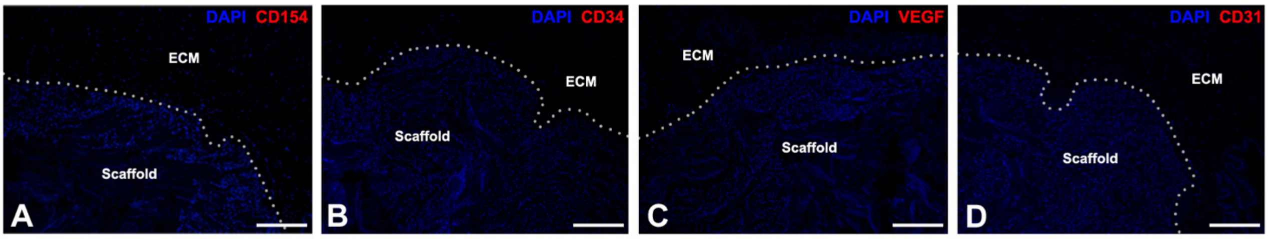

Figure S1. Immunofluorescence negative controls. Representative images of immunofluorescence assays performed by omitting the primary antibodies: CD154 (A), CD34 (B), VEGF (C), and CD31 (D). Only nuclear staining with DAPI is visible, confirming the absence of nonspecific bindings. Dashed lines delineate the boundaries between scaffold and ECM. ECM: extracellular matrix. Bars: 50 μm.

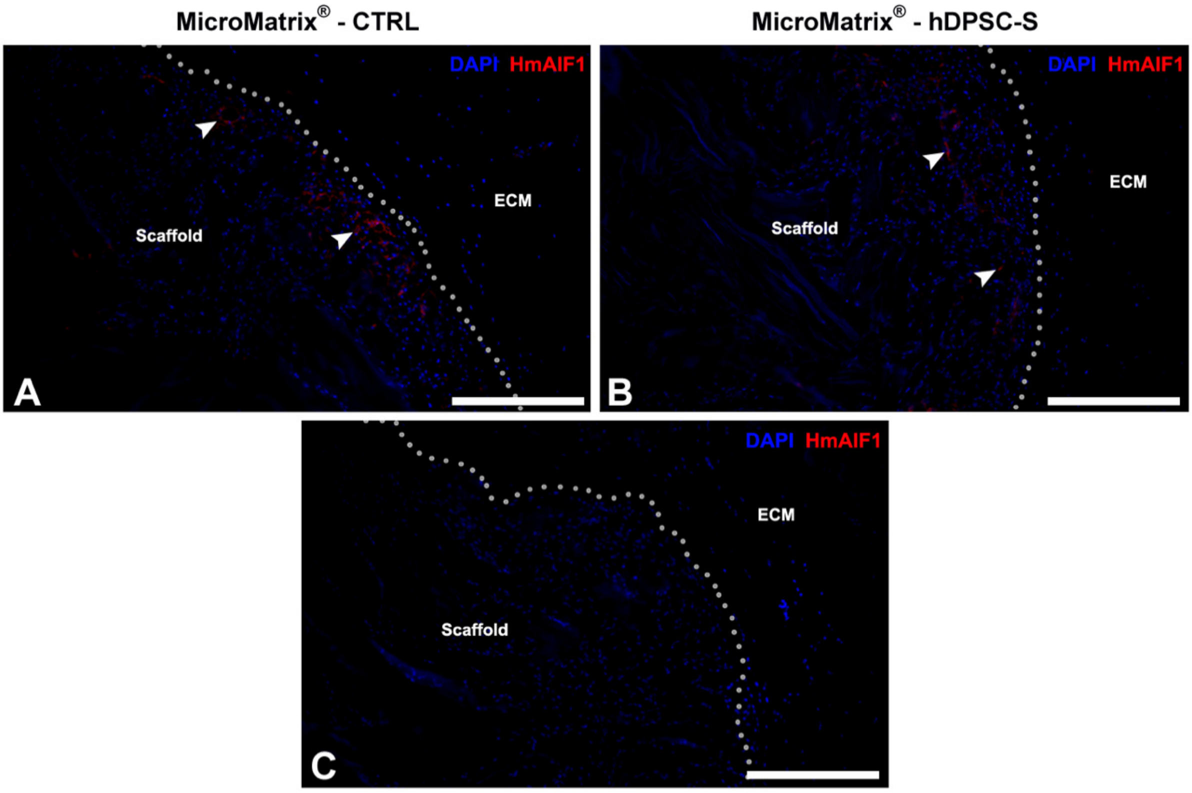

Figure S2. Immunofluorescence assay for *Hm*AIF1. In both experimental conditions (A,B), a weak *Hm*AIF1 positivity (arrowheads) is observed at the interface between the scaffold and the surrounding extracellular matrix (ECM). In the negative control (C), in which the primary antibody was omitted, no red signal is detected. Dashed lines delineate the boundaries between scaffold and ECM. ECM: extracellular matrix. Bars: 50 μm.
